# Osteosarcoma in a Teenage Athlete With a Swollen Knee Joint

**DOI:** 10.7759/cureus.56366

**Published:** 2024-03-18

**Authors:** Amresh Gul, Zahid Khan

**Affiliations:** 1 General Practice, Lifeline Hospital, Salalah, OMN; 2 Acute Medicine, Mid and South Essex NHS Foundation Trust, Southend on Sea, GBR; 3 Cardiology, Bart’s Heart Centre UK, London, GBR; 4 Cardiology and General Medicine, Barking, Havering and Redbridge University Hospitals NHS Trust, London, GBR; 5 Cardiology, Royal Free Hospital, London, GBR

**Keywords:** bone malignancy, malignant bone tumor, lytic bone lesion, osteosarcoma knee, osteosarcoma research

## Abstract

Osteosarcoma is a malignant mesenchymal tumour. This primarily manifests in the formation of immature osteoid cells by tumour cells. Osteosarcoma is the most common primary bone tumour in children and adolescents. It tends to occur in the metaphysis of long shafts, shows osteoblastic differentiation, and produces malignant osteoid material. We present the case of a 17-year-old male who presented to our clinic who had left knee pain for a few days. An initial radiograph of the knee joint revealed a lytic lesion in the proximal tibia and further imaging was advised. During a follow-up visit, the patient had worsening pain and had a computerized tomography scan of the left knee, confirming osteosarcoma on the lateral side of the left tibia. He was referred to the orthopaedic department, where a biopsy was performed, to confirm the diagnosis of osteosarcoma. The patient was commenced on chemotherapy due to metastatic disease and has so far tolerated therapy well.

## Introduction

Osteosarcoma is a primary malignant tumour of the bone that is caused by proliferating neoplastic cells that, albeit in small amounts, produce osteoids and bone. This histological principle describes a tumour that disproportionately affects the long bones of the appendicular skeleton and typically affects young males more often than females. These tumours typically have a high degree of local aggressiveness and frequently develop early fatal systemic metastases [1,2,3]. Numerous patients initially report experiencing intermittent pain that varies in intensity, may be worse at night, and may persist for a considerable amount of time [3]. Adolescent athletes frequently report pain in the lower femur, the area just below the knee. Large tumours may manifest as an obvious localized swelling [3,4]. Because the damaged bone is weaker than the healthy bone and is prone to abnormal fractures from small trauma, the first sign of an illness can occasionally be a sudden fracture [5,6]. Localised swelling might not be noticeable in tumours that are deeper and farther away from the skin, such as those that start in the pelvis [6].

The human sarcoma gene expression profiling analysis showed closely clustered groups, which may indicate that each branch shares common signalling pathways [7]. From a molecular perspective, these neoplasms are classified into two main groups. The first group is sarcomas with specific genetic mutations (c-kit in gastrointestinal stromal tumours) and translocations that result in genes fusion (for example, EWS/FLI-1 (Ewing's sarcoma/Friend leukemia integration site 1) fusion gene in Ewing sarcoma), and the second group is sarcomas with non-specific gene alterations including very complex karyotypes with numerous gains and losses [8,9].

Osteoblastic cells in this area are more likely to proliferate and carry mutations that may cause cell transformation, which is why osteosarcomas typically develop at the sites of bone growth (retinoblastoma protein (RB1) and p53 genes are commonly involved). The tumour may be located at the epiphysis, which is at the end of the long bone [10,11]. It typically affects the distal end of the femur, the proximal end of the tibia, or the humerus. Osteosarcoma usually affects the area around the knee in almost 60% of cases followed by the hip accounting for 15%, the shoulder in 10%, and the jaw in about 8%. The tumour mostly appears solid, hard, and irregular on X-ray examination ("fir-tree”, "moth-eaten”, or "sun-burst" appearance) due to the tumour's calcified bone spicules radiating at right angles [11,12,13]. We present a case of osteosarcoma in a young teenager who presented with left knee pain and swelling for a few days. 

## Case presentation

A 17-year-old male presented to our clinic with a few days of severe pain in the left knee. He described the pain as severe, throbbing, localised, and had worsened over the past 72 hours. This was accompanied by worsening progressive swelling of his left knee over the past 48 hours. He was limping and was unable to tolerate weight-bearing. His vital signs were stable, and on examination, he had swelling of the left knee and was tender to touch. The remaining physical examination was unremarkable. There was no significant past medical history of note and he was not on any regular medications.

**Figure 1:**
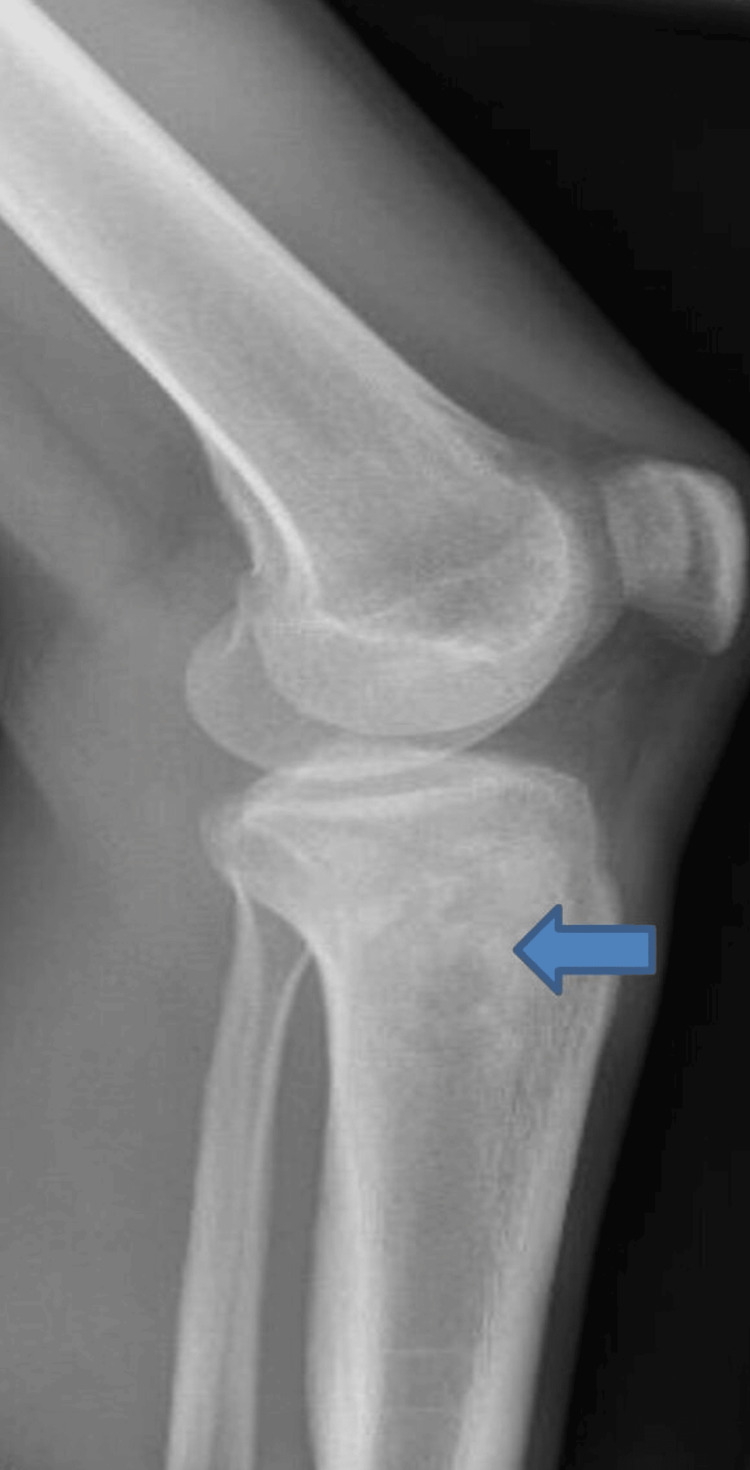
X-ray of the left knee showing lytic lesion (Blue arrow)

An initial X-ray showed the aggressive mixed sclerotic and lytic lesion at the lateral aspect proximal tibial metaphysis with aggressive periosteal reaction and surrounding soft tissue ossification (Figure 1). He received initial treatment with analgesics including meloxicam and cocodamol. Two days later, he presented to his general practitioner with worsening pain and swelling and was referred to the local hospital Accident and Emergency Department, where he was reviewed by the trauma and orthopaedic team. The patient was administered analgesia and booked for an urgent outpatient computerized tomography (CT) scan of the left knee. His laboratory results are shown in Table 1.

**Table 1:** Laboratory results for patients on days 1 and 28.

Test	Day 1	Day 28	Reference value
Sodium	143	141	135-145 mmol
Potassium	3.9	4.7	135-145 mmol
Chloride	105	106	96-106 mmol
Bicarbonate	30	30	22-32 mmol
Glucose	4.9	5.2	3.9 - 5.6 mmol
Urea	6.2	6.5	2.1 - 8.5 mmol
Creatinine	99	106	53- 110 mmol
Urate	0.37	0.37	0.24 - 0.51 mmol
Protein (total)	55	58	60 – 83 g/L
Albumin	31	33	35 – 45 g/L
Globulin	21	25	20 – 35 g/L
Bilirubin (Total)	5	5.5	0 - 21 umol/L
Bilirubin (Conjugated)	<4	<4	0 – 5 umol/L
Alkaline phosphatase	112	117	30–130 U/L
Gamma-glutamyl transferase	128	129	0- 30 U/L
Aspartate transaminase	40	43	0- 40 U/L
Alanine transaminase	165	169	04 - 36 U/L
Lactate dehydrogenase	232	211	140 - 280 U/L
Calcium	2.26	2.29	2.2 - 2.6 mmol
Phosphate	1.34	1.38	0.81 - 1.45 mmol
Magnesium	.68	0.68	0.65 - 1.05 mmol
Haemoglobin	96	93	135 - 180g/L
White cell count	5.1	5.3	3.5 – 11 x10^9^/L
Platelet count	217	220	140 - 400 x10^9^/L
Mean corpuscular haemoglobin	24.9	25.1	27 - 33 pg
Mean corpuscular volume	79	77	80 - 100 fL
Neutrophils	3.4	3.35	2 - 8 x10^9^/L

**Figure 2:**
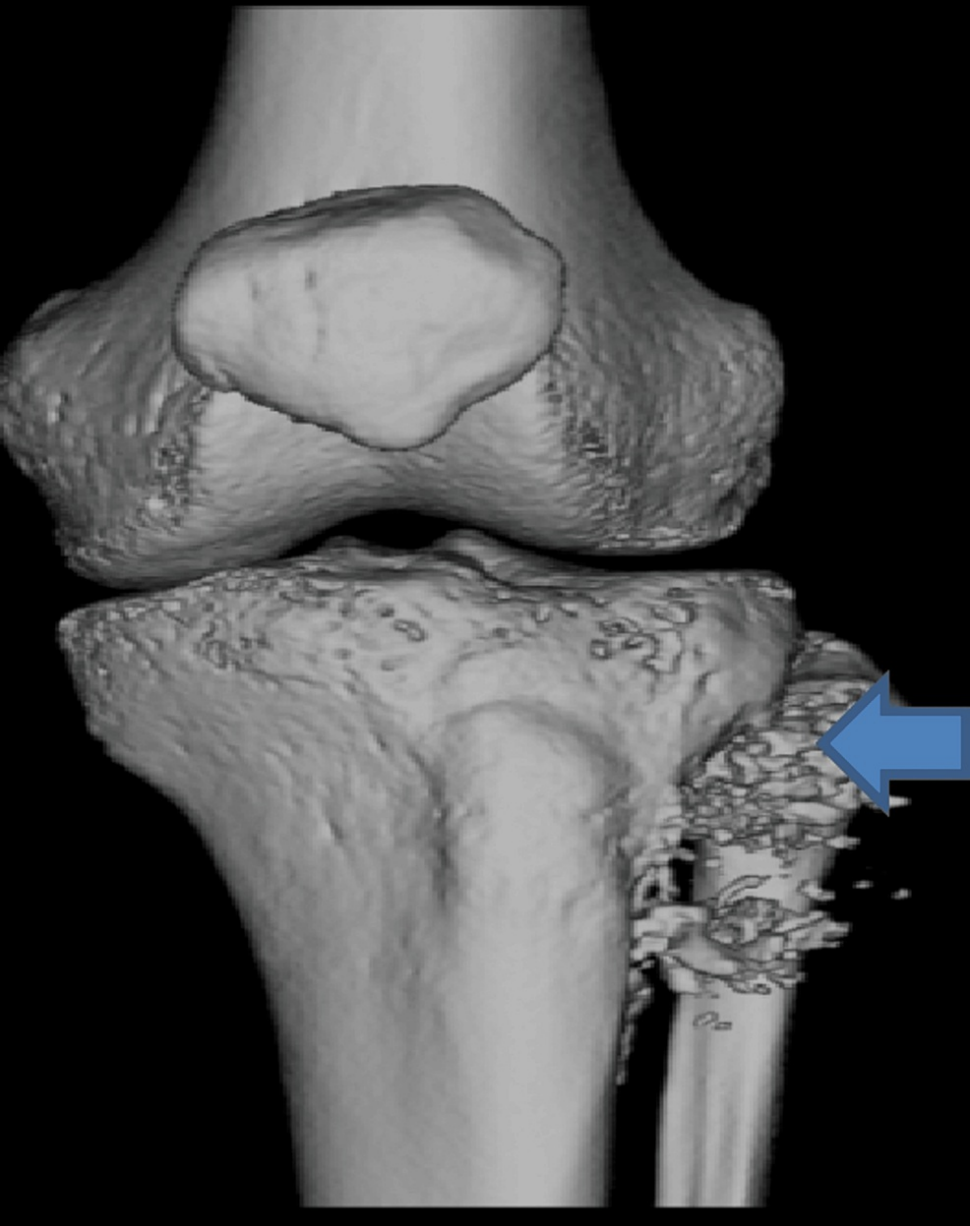
Computerized tomography scan of the left knee showing osteosarcoma and bony erosion (Blue arrow)

**Figure 3:**
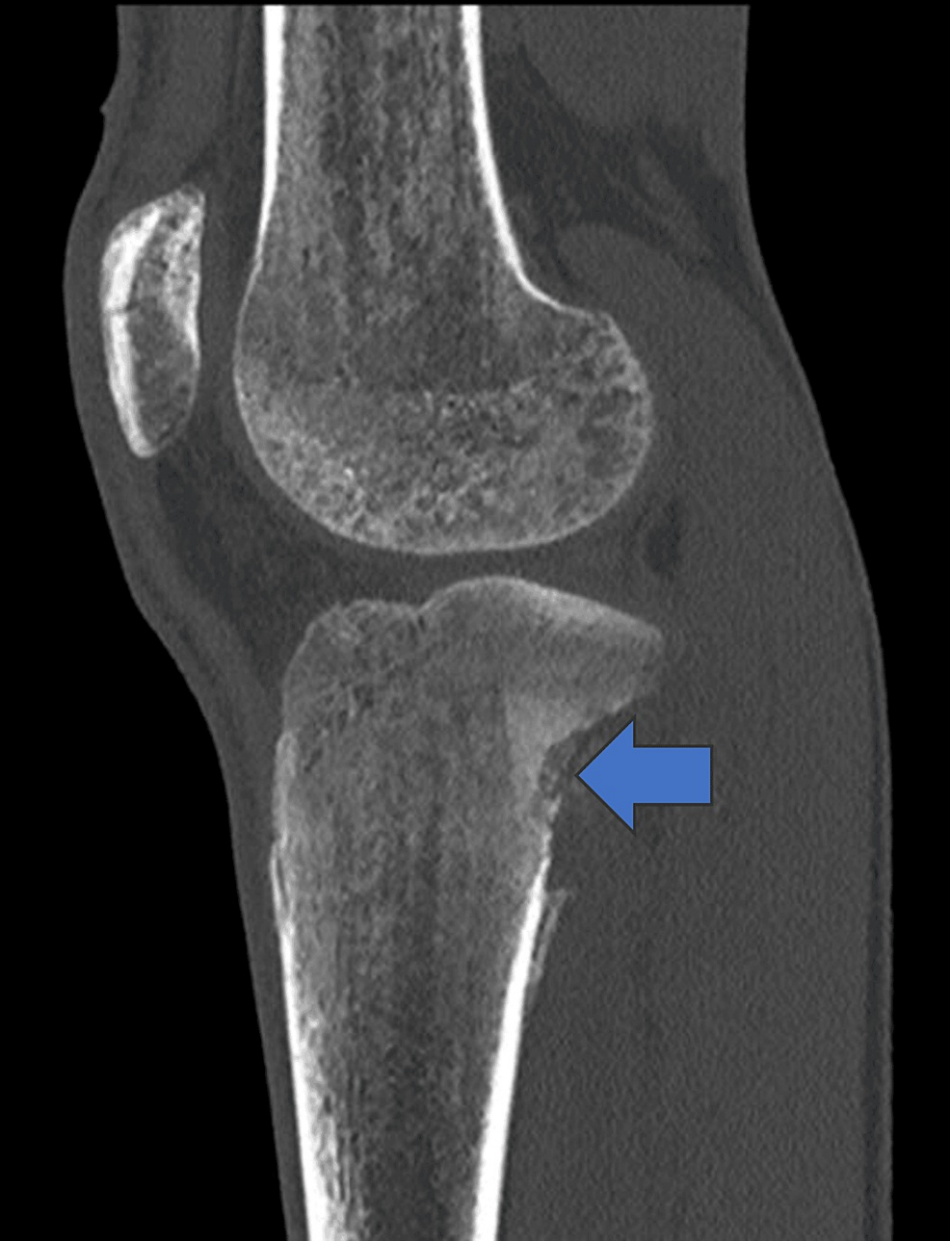
Left knee/tibial osteosarcoma on computerized tomography scan (Blue arrow)

The CT scan of the left knee, upper tibia and fibula revealed an aggressive bone lesion seen as an area of permeative bone destruction in the upper posterolateral tibial meta diaphysis extending to the subchondral location with cortical break and associated soft tissue mass measuring approximately 4.6 x 2.8 cm with aggressive periosteal reaction in the form of Codman triangles and soft tissue ossifications (Figures 2, 3). There was extensive marrow oedema and marrow replacement in the fibular head concerning for further tumor infiltration extending approximately 90 mm below the tibial plateau at the same level to the most inferior margin of the soft tissue component resulting in the widening of the proximal tibiofibular joint with posterior displacement of the popliteus muscle and popliteal sheath bundles. The mass approximates the tibiofibular trunk and partial overbreach of the popliteus muscle. The patient was discussed in a multidisciplinary meeting and a biopsy was recommended due to suspicion of the lesion being osteosarcoma. Percutaneous biopsy revealed a malignant tumour composed of nests of epithelioid cells with eccentrically placed pleomorphic nuclei. CT thorax, abdomen and pelvis (CT-TAP) demonstrated a rounded 3 mm pulmonary nodule in the right lateral lower lobe which could be non-specific or metastatic in settings of osteosarcoma and further small ground glass nodules were noted in the left lower lobe suggestive of infection/inflammatory process, noting moderate volume retains secretions in the left lower lobe bronchus. A further lesion was identified in the liver suspected to be malignant and bilateral groin lymphadenopathy was noted on a positron emission computerized tomography scan (PET-CT).

The tumour cells stained positive for AT-rich sequence-binding protein 2 (SATB2). Cytoplasmic and membranous staining for CD99 was positive. P16 immunohistochemistry showed diffuse strong, diffuse positive staining. Cytokeratin AE1/AE3, S100 protein, SRY (sex-determining region Y protein)-related HMG-box 10 protein (SOX0), WT-1desmin, myogenin factor MYF4, and ERD were negative. Nuclear staining for friend leukemia integration 1 transcription factor (FLI-1) was positive, suggestive of small cell osteosarcoma. The patient was found to have bilateral lymphadenopathy in the groin. The patient did not have surgery due to metastatic lymphadenopathy and suspected liver lesions and was commenced on methotrexate, folinic acid, and leucovorin infusion during hospitalization. Currently, he is receiving chemotherapy with cisplatin, doxorubicin, methotrexate, and leucovorin (calcium folinate) and has tolerated therapy well. He remains under follow-up and is responding well to treatment apart from having the usual side effects of nausea and fatigue.

## Discussion

Osteosarcoma is a primary malignant tumor of the bone that occurs more commonly in men [4]. Incidence rates and 95% confidence intervals for osteosarcoma in all races and sexes are 4.0 (3.5 to 4.6) per million people per year in the age range 0 to 14 years and 5.0 (4.6 to 5.6) in the age range 0 to 19 years [14]. This cancer has a bi-age distribution, with a first peak in adolescence and a second peak in adulthood. The first peak occurs between the ages of 10 and 14, coinciding with the rapid development of puberty [14]. Osteosarcoma primarily occurs in the knee (60%), hip (15%) and about 10% near the humerus. The main pathophysiological mechanisms include several that may contribute to bone formation-related genetic disorders that lead to malignant progression and metastasis [15].

Most patients with osteosarcoma of the extremities complain of pain prior to soft tissue swelling. This is true of any primary bone tumor because stretching of the periosteum often causes pain before the tumor becomes apparent [6]. Pain can also be due to bone thinning and volume loss resulting in small stress fractures. The appearance of sudden and severe pain signals a significant pathological fracture, which is rare in adult patients [16]. Up to 15% of paediatric patients experience pathological bone fractures. The second most common complaint is swelling associated with soft tissue masses. Although approximately 90% of osteosarcomas spread to soft tissues, some patients complain of swelling. Systemic symptoms, such as weight loss, pallor, fever, and anorexia, are rare. [17,18].

Radiological examination is the standard examination method for all patients with bone abnormalities. Large lesions can be found in patients with osteosarcoma and are mainly manifested by the destruction of normal bone trabeculae with blurred edges. Lesions often stimulate new bone formation at the periosteum, creating the characteristic Codman triangle [6,17]. For a more detailed evaluation, CT or magnetic resonance imaging (MRI) may be performed. A CT scan allows a detailed evaluation of most bone tumors. MRI can better delineate the involved soft tissue masses, which can effectively inform subsequent biopsies and final surgical resections. MRI often captures skip metastases from local tumors [19,20].

Our study highlights the importance of early treatment and accurate diagnosis of osteosarcoma. Surgical treatment with modular allograft reconstruction of osteosarcoma allows limb salvage and does not affect quality of life. Surgery and the combination of chemotherapy in therapy have proven to be effective, however, surgery may not be possible in advanced or metastatic lesions [19,20].

## Conclusions

Due to the early diagnosis of bone cancer and no metastases by surgical resection, the so-called limb conservation treatment can be applied in all cases due to modular prosthetic reconstruction. Surgical treatment and adjuvant chemotherapy make this pathology a good prognostic index and do not worsen quality of life. We conclude that the function of the operated limb never reaches the same level of function as the normal head; however, the quality of life does not change significantly, allowing the patient to perform normal daily activities. It should be emphasised that the main goal of the general treatment of bone cancer is to provide longer recurrence-free survival, and only the second time the goal is to preserve limb function.
